# Identification of readily available pseudo-natural products†

**DOI:** 10.1039/d4md00310a

**Published:** 2024-07-04

**Authors:** Axel Pahl, Oleksandr O. Grygorenko, Ivan S. Kondratov, Herbert Waldmann

**Affiliations:** a Compound Management and Screening Center (COMAS), Max Planck Institute of Molecular Physiology Otto-Hahn-Strasse 11 44227 Dortmund Germany; b Enamine Ltd. Chervonotkatska Street 78 Kyïv 02094 Ukraine https://enamine.net; c Taras Shevchenko National University of Kyiv Volodymyrska Street 60 Kyïv 01601 Ukraine; d V.P. Kukhar Institute of Bioorganic Chemistry & Petrochemistry, NAS of Ukraine Akademik Kukhar Street 1 Kyïv 02660 Ukraine; e Enamine Germany GmbH, Industriepark Hoechst G837 65926 Frankfurt am Main Germany https://www.enamine.de; f Department of Chemical Biology, Max Planck Institute of Molecular Physiology Otto-Hahn-Strasse 11 44227 Dortmund Germany herbert.waldmann@mpi-dortmund.mpg.de; g Faculty of Chemistry and Chemical Biology, TU Dortmund University Otto-Hahn-Strasse 6 44221 Dortmund Germany

## Abstract

Pseudo-natural products (PNPs) combine fragments derived from NPs in ways that are not found in nature, and may lead to the discovery of novel chemotypes for unexpected targets or the identification of unprecedented bioactivities. PNPs have increasingly been explored in recent drug discovery programs, and are strongly enriched in clinical compounds. We describe how a large number of structurally different PNPs can be accessed readily and without the need to execute labor- and time intensive synthesis programs. We employed an improved version of the previously reported natural product fragment combination (NPFC) tool to analyze the full library of 3.5 M synthetic small molecules and screening libraries from Enamine for PNP content, assessed the spatial complexity of Enamine-PNPs using the recently developed normalized spatial score (nSPS) and evaluated the bioactivity of a selected subset of Enamine-PNPs in the unbiased morphological cell painting assay. A major fraction (32%; 1.1 million compounds) of the Enamine library are PNPs which contain a significant number of compounds with unexpected and probably new bioactivity.

## Abstract 2

Pseudo-natural products (PNPs) combine fragments derived from NPs in ways that are not found in nature, and may lead to the discovery of novel chemotypes for unexpected targets or the identification of unprecedented bioactivities.

## Graphical abstract

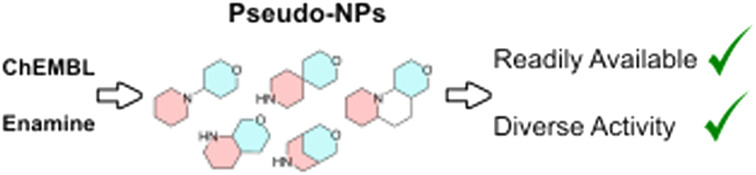

## Introduction

The structures of compounds with proven relevance to nature have fuelled numerous research and development programs aimed at the discovery of novel bioactive small molecules. Thus, natural products (NPs) created in evolution have been and continue to be a rich source of drugs,^1^ and they have inspired different principles for bioactive small molecule discovery^2,3^ including diversity-oriented synthesis,^4^ the complexity-to-diversity approach,^5^ and biology-oriented synthesis.^6^ In light of the limited coverage of biologically relevant chemical space explored in evolution, we have recently introduced the pseudo-natural product (pseudo-NP; PNP) concept.^7–9^ The PNP strategy combines evolutionary logic with synthetic chemistry to accelerate exploration of NP-like chemical space, and, to this end, pseudo-natural products combine fragments derived from NPs or fragment-sized NPs in ways that are not found in nature. They thereby escape the structural boundaries of current biosynthetic pathways and expand coverage of NP-like chemical space not populated by NPs, while pertaining their properties, in particular biological relevance. PNP collections were typically synthesized in 2–5 step reaction sequences which frequently include asymmetric complexity-generating transformations often forming multiple stereogenic centers. Investigation of pseudo-NP collections may lead to the discovery of novel chemotypes for unexpected targets or the identification of unprecedented bioactivities. For instance, we identified novel pseudo-NP inhibitors of glucose transporters,^10^ kinases,^11,12^ and the first inhibitors of the cholesterol-shuttling protein GRAMD1A^13^ and the Rho-protein chaperone Rho-GDI.^14^

In order to unravel whether the synthesis and biological evaluation of PNPs might have been explored in a wider sense before, for instance driven by intuitive inclusion of NP structures in compound library design, we recently developed the natural product fragment combination (NPFC) tool. This tool allows the identification and classification of PNPs in large compound collections by identifying NP fragments and their combination types in chemical structures. Indeed, application of the NPFC tool to the ChEMBL database, v26 (ref. 15) revealed that PNPs have been synthesized and investigated for bioactivity before widely, and that PNPs define a major fraction of bioactive compounds as described in the ChEMBL database.

Application of an updated version of this methodology (see below) to clinical compounds in phases 1–3 and 4 (*i.e.* on the market) very recently demonstrated that PNPs are strongly enriched in clinical compounds.^16^

In light of this broad historical use and success, rapid access to PNPs, preferably without the need to develop multi-step complexity-generating asymmetric transformations, would be of high relevance to future medicinal chemistry and chemical biology programs.

In order to devise a way to get rapid access to a large number of structurally different PNPs, we analyzed the full library of 3.5 million synthetic small molecules and screening libraries, which are available and widely sourced by the scientific community from Enamine^17,18^ for PNP content (Enamine-PNPs), as a proxy for potential future bioactive compounds. We assessed the spatial complexity of the Enamine library using the recently developed normalized spatial score (nSPS),^19^ and evaluated the bioactivity of a selected subset of PNPs obtained from Enamine in the unbiased morphological cell painting assay (CPA). The analysis revealed that 32% of the Enamine library are PNPs. Their predominant fragment composition and combinations resemble those of PNPs identified in the recently released ChEMBL 32 dataset. We also show that Enamine-PNPs contain a significant number of compounds with unexpected and probably new bioactivity.

## Results and discussion

The NPFC tool identifies natural product fragments^20^ present in the investigated compounds and analyzes their combination types by generating fragment combination graphs (FCGs). By comparing these graphs to those present in NPs,^21^ the tool classifies a given structure as either PNP, when it has FCGs that are not found in NPs; natural product like (NPL), when it has FCGs that are also present in NPs; NP, when the structure is identical to a NP; and finally, NonPNP for compounds that do not match any of these categories. When a compound has both PNP and NPL FCGs, it is classified as a PNP. Although the NPFC tool applies several structural filters, like number of rings >0 and molecular weight < 1000, and deduplicates racemic structures after standardization by InChIKey before performing the analysis, parsing the output of the tool enables assignment of PNP-, NPL-, NP- and NonPNP status to a given set of compounds.

In order to enable an up-to-date analysis of currently investigated bioactive compounds, we applied the NPFC tool to the latest version of the ChEMBL database (v32, released in 2023; 2.3 million compounds) as described in the experimental section. In the course of this analysis, it was found that the previously described version of the tool^15^ erroneously removed FCG duplicates when they were present in the same computing chunk, resulting in a significant under-representation of PNPs. Therefore, we report here the updated numbers for ChEMBL v32, confirming the expected increase of numbers for PNPs.

**Fig. 1:**
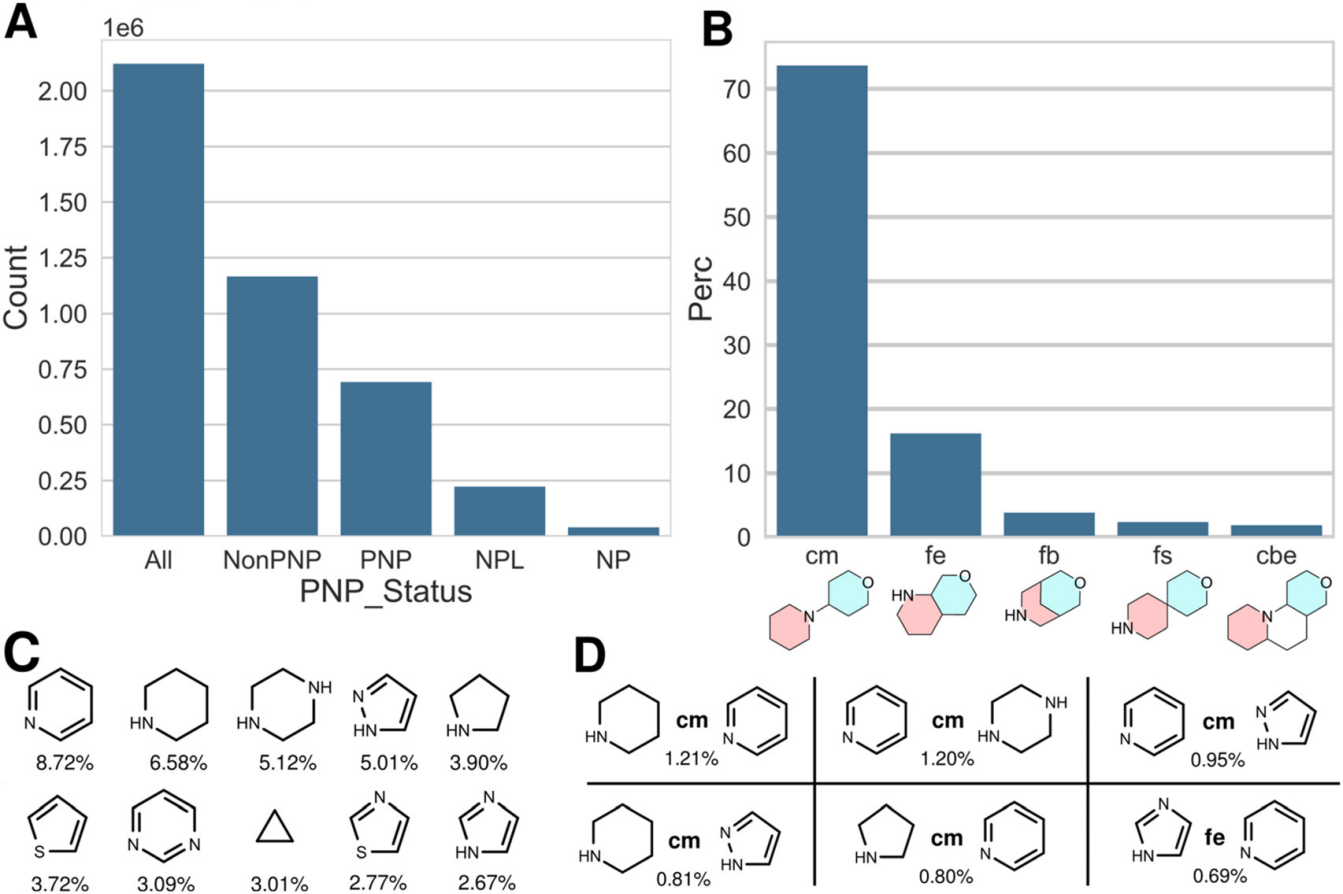
A: Distribution of PNP status for the ChEMBL v32 dataset, deduplicated by InChIKeys (*y*-axis scale in million compounds). B–D: Data for PNP compounds, only. B: Distribution of most common NP fragment connection types in percent; cm: connection monopodal, fe: fusion edge, fb: fusion bridge, fs: fusion spiro, cbe: connection bipodal edge. C: Most common NP fragments, with percent occurrence in all PNP NP fragment combinations. D: Most common NP fragment combinations, with connection type and percent occurrence (see B for explanation of connection types).

Fig. 1 shows the results obtained for the ChEMBL v32 dataset, deduplicated by InChIKeys of the standardized and racemic structures (2.1 million compounds). Within that dataset, 690 000 PNPs were identified, which is 32% of the listed bioactive compounds. In the analysis provided earlier,^15^ 340 000 PNPs had been identified, *i.e.* the fraction of PNPs in the ChEMBL database actually is significantly higher than previously estimated. In addition, 220 000 natural product-like compounds (NPLs) and 39 500 natural products (NPs) were identified (Fig. 1A). These numbers differ from the previously determined results, where 35 000 NPs were identified; the number of NPLs was not determined in our previous report.

By analogy to the previously reported analysis, the most frequent connection between two NP fragments in ChEMBL-PNPs is the linear connection (connection monopodal (cm)), followed by an edge fusion (fusion edge (fe)), a bridge fusion (fb), a spiro fusion (fs) and the bipodal edge connection (cbe; for a graphical representation of the different fusion and connection types, see Fig. 1B and ref. 15). The relative distribution of the different connection and fusion types is cm : fe : fb : fs : cbe ≈ 100 : 22 : 5 : 3 : 2.5. Fig. 1C shows the most abundant fragments found in PNPs, while Fig. 1D displays the most common combination of fragments, including their connection types.

These data show that the conclusions drawn previously in the analysis of ChEMBL v26 employing the initial version of the NPFC tool remain valid and are actually enforced after application of the improved NPFC tool.

For future widespread application and exploration of PNPs in medicinal chemistry and chemical biology programs, uncomplicated and fast access to different PNP classes without the need to launch and execute lengthy synthesis programs would be instrumental. The Enamine collection of currently *ca.* 3.5 million compound collection is a rich source of structurally diverse small molecules and is frequently sourced for research and discovery by industry and academia. Given the fact that PNPs have been synthesized frequently before (see above), probably driven by intuitive inclusion of NP structures in compound library design, we speculated that this very large library also might contain a substantial number of PNPs.

**Fig. 2:**
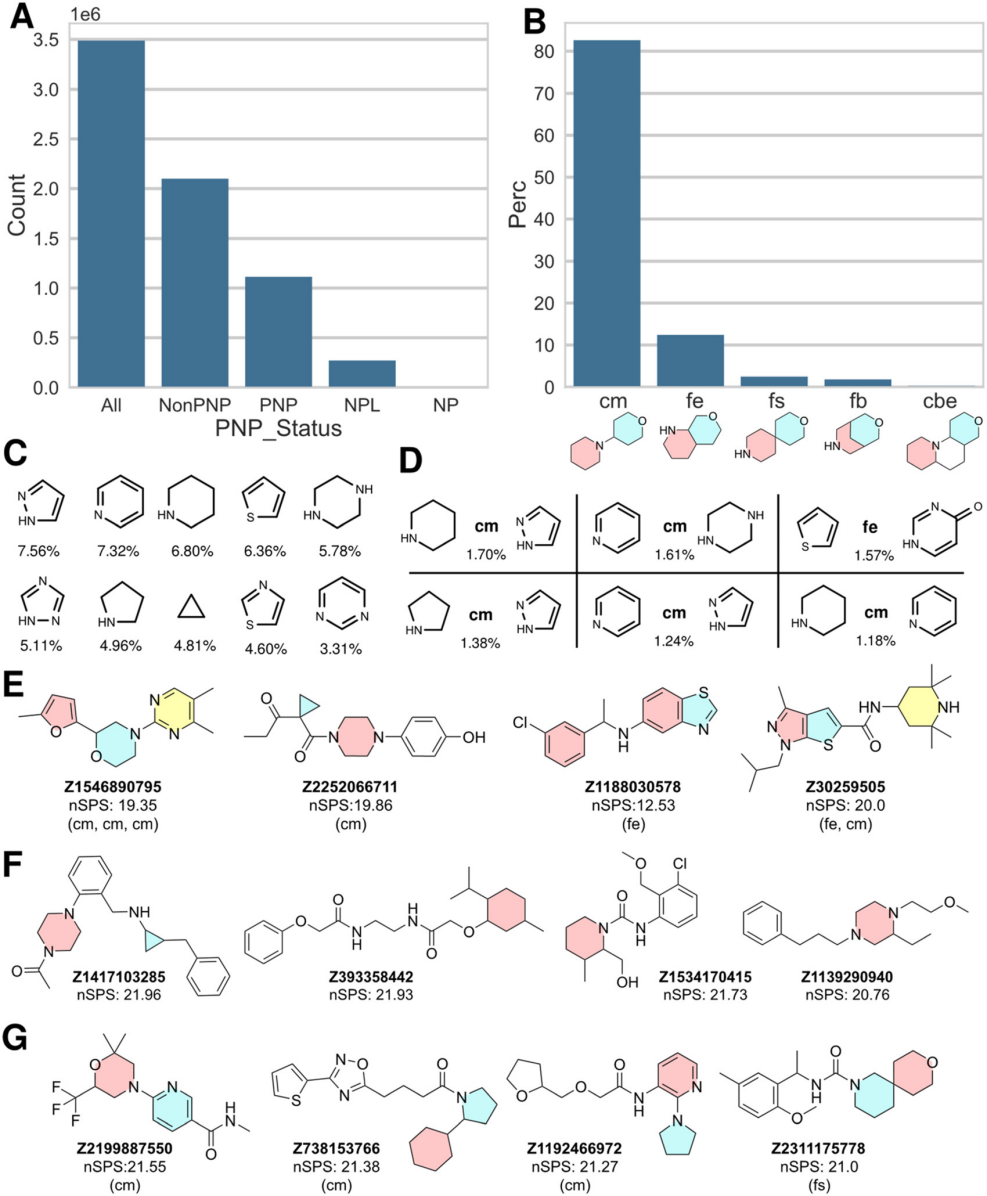
A: Distribution of PNP status for the full Enamine dataset (*y*-axis scale in million compounds). B–D: Data for PNP compounds, only. B: Distribution of most common NP fragment connection types in percent; cm: connection monopodal, fe: fusion edge, fs: fusion spiro, fb: fusion bridge, cbe: connection bipodal edge. C: Most common NP fragments, with percent occurrence in all PNP NP fragment combinations. D: Most common NP fragment combinations, with connection type and percent occurrence (see B for explanation of connection types). E–G: Example structures; E: PNP, F: NonPNP, G: NPL.

In order to explore this notion, we analyzed the structures of the Enamine library dataset with the NPFC package^22^ and merged the PNP status information and the fragment combination information to the original full dataset. The resulting classification revealed that the Enamine library contains 32% PNPs (1.1 million in total and after deduplication by InChIKey), which is remarkably similar to the result obtained for the ChEMBL dataset. 270 000 NPLs and 4000 NPs were also identified. All other compounds were denoted by a “Non-PNP” status (Fig. 2A). Fig. 2E shows examples of PNPs identified in the Enamine library, *i.e.* Enamine-PNPs.

As in the ChEMBL dataset, the linear connection (connection monopodal (cm)) is the most common connection type found for the fragment combinations within the Enamine-PNPs (83%), followed by fusion edge (fe; 12%), fusion spiro (fs; 2.5%), fusion bridge (fb; 1.8%) and connection bipodal edge (cbe; 0.3%). The relative distribution of the different connection and fusion types is cm : fe : fb : fs : cbe ≈ 100 : 15 : 3.0 : 2.2 : 0.1. Thus, in the Enamine library PNPs with monopodal connectivity are significantly more prevalent than in the ChEMBL dataset. This is due to synthesis reasons, since compounds with monopodal fragment connectivity are more straightforward to synthesize than for instance fragment combinations with spiro or bridged fusion. Fig. 2C and D show the 10 most common fragments found in the NP fragment combinations of the PNPs and the 6 most common fragment combinations, respectively. The full list of fragments and fragment combinations found in Enamine PNPs is available as ESI.†

To assess how the Enamine library in general, and the identified PNPs more specifically, compare to each other, we generated the following three datasets:

1. *Enamine-All*: The full set of Enamine synthetic compounds (3.5 M).

2. *Enamine-PNP*: The set of identified Enamine PNPs (1.1 M).

3. *Enamine-PNP CPA*: A representative subset of Enamine PNPs to be tested for bioactivity. To narrow down the selection, only compounds where the Murcko scaffold consisted of exactly 17 heavy atoms were considered, as this was determined to be the median value for the approved and experimental subsets of the DrugBank (v5.1.8).^23^ In addition, only scaffolds with 4 or more representatives were considered. From these, 250 scaffolds were chosen randomly and from each scaffold four compounds were in turn selected, again randomly. Finally, 875 compounds matching purity criteria (≥90% LCMS) were obtained.

**Fig. 3:**
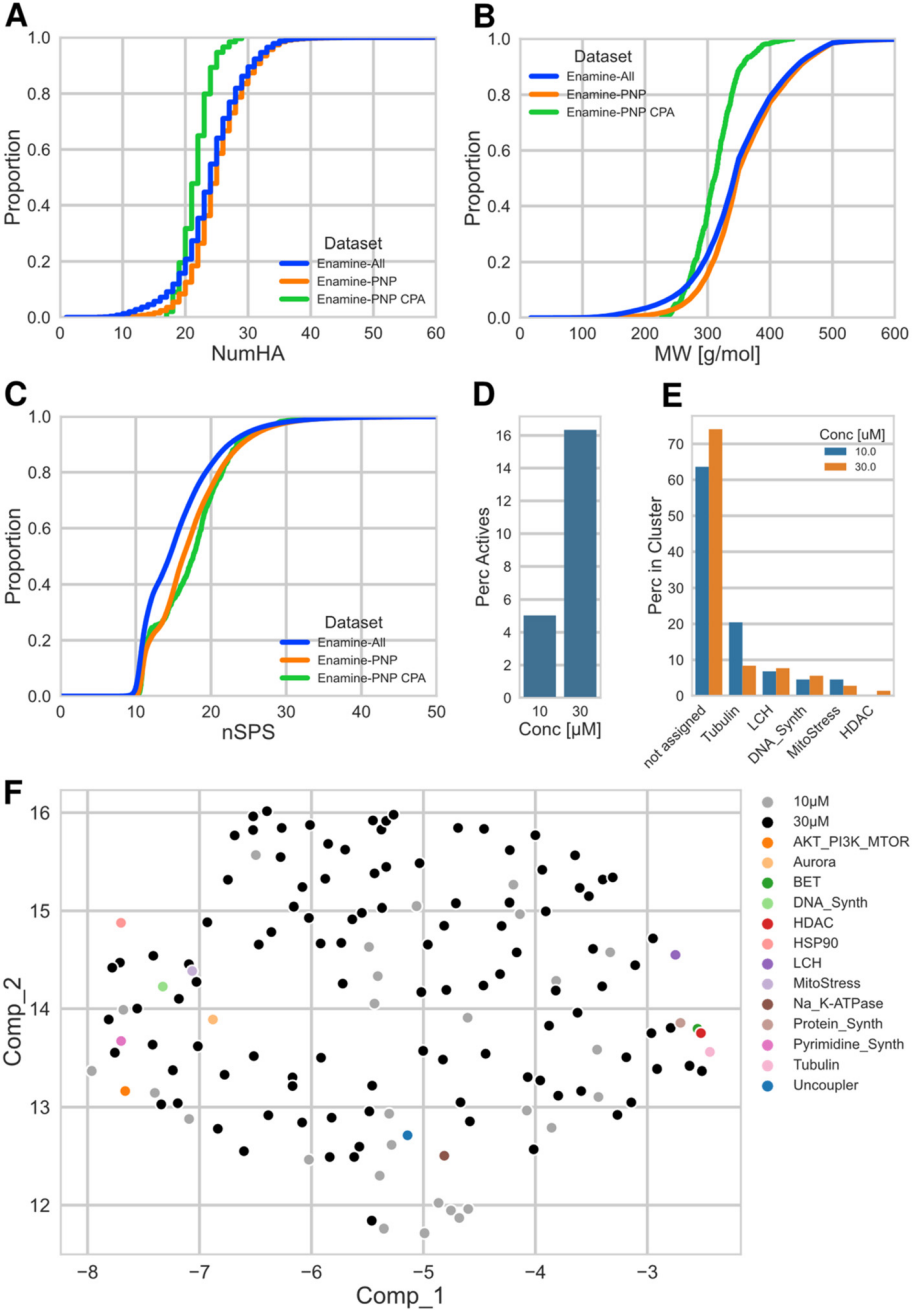
A: ECDF plot of number of heavy atoms; datasets: *Enamine-All*: full Enamine library of 3.5 M cpds, Enamine-PNP: 1.1 M PNPs identified by NPFC analysis, Enamine-PNP CPA: selected subset of 875 cpds. for testing in CPA. B: ECDF plot of the molecular weight; for description of datasets, see (A). C: ECDF plot of the normalized spacial score nSPS; for description of datasets, see (A). D: Percentage of active (≥5% induction) cpds. in the CPA, at 10 and 30 μM for the Enamine-PNP CPA subset. E: Assigned biological clusters for the cpds. active at 10 and 30 μM. F: UMAP plot using the full morphological profiles (579 features) of the 28 (10 μM)/106 (30 μM) cpds. that were not assigned to any of the 13 clusters; representative cpds. for each of the biological clusters have been added as markers for the phenotypic space.

Fig. 3(A–C) shows the distributions of number of heavy (non-hydrogen) atoms (NumHA), molecular weight (MW) and normalized spatial score (nSPS) for the three datasets as empirical cumulative distribution function plots (ECDF). nSPS is a recently introduced score to describe 3D (spatial) molecular complexity, taking into account hybridization, stereogenic centers, branching and ring membership of the constituting atoms. The score is generally normalized by the number of heavy atoms (nSPS) and consequently represents a complexity density.^19^

The distributions of the molecular weight show that approx. 80% of the full Enamine library and of the Enamine PNPs (*Enamine-All* and *Enamine-PNP*) have values below 400 g mol^−1^. This is mainly because of drug- and lead-likeness concepts prioritizing physicochemical properties of the compounds that were in the focus of the medicinal chemistry community in the 2000s and partially – in 2010s, and which still widely used today.^24^ In other words, the Enamine screening collection focuses on structures of smaller size, making the compounds suitable as primary hits that can be further functionalized and structurally evolved. This is also reflected by the distributions of NumHA. The distributions of NumHA and MW for the subset selected for CPA (*Enamine-PNP CPA*) are shifted towards smaller molecules. Compared to the larger collections, practically all compounds in the selected set have a MW ≤ 400 g mol^−1^. For the nSPS, the distribution of both, the *Enamine-PNPs* and the selected CPA subset are shifted to higher complexity scores, relative to the full Enamine library (*Enamine-All*). This could be hinting at a general higher molecular complexity of the PNP structures (medians of nSPS values: *Enamine-All*: 14.5, *Enamine-PNP*: 16.3, *Enamine-PNP CPA*: 17.6).

For unbiased assessment of compound bioactivity, we employed the cell painting assay (CPA). This multi-featured morphological assay enables the identification of a number of bioactivities and mechanisms of action (MoAs) without prior target hypotheses.^25^ It monitors bioactivity in a very broad sense and, has been proven to be a highly valuable method for comparison of bioactivity for PNPs with different fragment combinations, connectivities and stereoisomeric relationships.^26–30^ In particular, the CPA enables identification of compound classes with diverse bioactivity differing from established small molecule classes.

Briefly, treated and untreated U2OS cells are stained with 6 different stains that are specific for different compartments of the cell, and imaged in 5 different channels by microscope. The images are then processed with CellProfiler^31^ and further analyzed using in-house written scripts during which the single-cell data is aggregated to compound level. The extracted morphological features are normalized to DMSO controls using a modified *Z*-score and the final generated profiles are investigated for their similarity to a set of pre-defined 13 clusters using a subprofile analysis.^32,33^ Details of the assay are described in the ESI.† The degree of bioactivity in the CPA is represented by the Induction value, *i.e.* the number of significantly changed features compared to DMSO controls, relative to the length of the full morphological profile, expressed in percent. Compounds with an Induction of ≥5% are considered to be active.

Fig. 3D shows the percent of active compounds in the Enamine-PNP subset that was submitted to the CPA. Gratifyingly, the subset contained a significant number of active measurements (CPA induction ≥ 5%), 44 (5%) and 143 (16%) of the tested 875 compounds were active at the two investigated concentrations, 10 μM and 30 μM.

We previously introduced a cluster subprofile analysis which allows the classification of active compounds into currently 13 bioactivity clusters based on activity in the CPA.^32,33^ Application of this cluster analysis to the Enamine-PNPs which had displayed bioactivity in CPA at the two concentrations revealed that among the identified clusters lysosomotropism/cholesterol homeostasis, tubulin modulation and DNA synthesis inhibition are the most prominent activities (Fig. 3E). The active Enamine-PNPs investigated here did not contain members that could be assigned to the Akt/PI3K/MTor, Aurora kinase, HSP90, Na^+^/K^+^-ATPase, protein synthesis, pyrimidine synthesis, and uncoupler clusters. Interestingly, 106 of the 143 Enamine-PNPs active at 30 μM (74%) could not be assigned to any cluster with a subprofile similarity of ≥80% (at 10 μM, 28 out of 44 active compounds (64%) could not be assigned). This finding might indicate that the PNPs identified in the Enamine compound set feature novel or unexpected bioactivity that differs from the reference compounds employed in the analysis. In order to analyze and visualize this promising finding, we performed a uniform manifold approximation and projection (UMAP^34^) analysis with the full morphological profiles (containing 579 features) of these unassigned PNPs and included compounds representing the 13 previously identified bioactivity clusters as markers for the corresponding phenotypic space. The scatter plot shown in Fig. 3F illustrates that the set of Enamine-PNPs active in CPA represents a broad biological activity. Very notably, the bioactivity of the novel PNPs differs from and goes beyond the activity of the reference compounds with annotated targets and activity. This finding highlights a qualitative advantage of PNPs and demonstrates that PNPs may be rich sources of compounds with unexpected and diverse, possibly previously unidentified bioactivity.

This finding shows that the unbiased synthesis of pseudo-NPs and their exploration in assays that broadly cover biological space may lead to the discovery of novel chemotypes with unexpected or novel bioactivities.

The CPA hit rate of 5% at 10 μM observed here for the *Enamine-PNP CPA* selection is lower than the corresponding value observed on average for COMAS in-house projects and reference compounds (hit rate: 31%). This difference can be explained by the properties of the Enamine collection.

As mentioned above, the design of the Enamine screening collection took into consideration drug- and lead-likeness concepts prevalent in drug discovery in the 2000s and partially – in 2010s, focusing on generating high-quality hits. As such, the molecule sizes and complexities are in the lower ranges (Fig. 3A–C), enabling modification and decoration of identified hits and also allowing for molecular weight increase during hit to lead and lead optimization programs.

We have shown recently a correlation of target-based activity with nSPS scores between 20–40,^19^ and demonstrated for selected PNP classes enriched in bioactivity that for them the nSPS also falls into this range.^35^ However, there is no direct correlation between the nSPS and activity in the CPA employed here as one of many possible measures of bioactivity, probably because of the unbiased nature of the assay and the experience that it also detects non-target related compound activity, like *e.g.* lysosomotropism and cholesterol homeostasis impairment.^36^ Still, an nSPS in the range of 20–40 will probably be advantageous. In addition, based on findings with our in-house data, there is a positive correlation between molecular weight and the hit rate in CPA (Fig. S1 and S2b† from ref. 30). These two findings together may explain the hit rate observed for the selected Enamine-PNPs. Indeed, although molecular weight has been identified before as a limiting property in the performance of compound collections,^37^ the design of the Enamine collection does reflect the generally higher demand of the medicinal chemistry community for compounds with lower molecular weight, which is also reflected in the compounds selected here for biological analysis.

In the selection of the compound collection investigated here, we had not taken nSPS or molecular weight into consideration. We now assume that a selection of PNPs with higher molecular weight and/or nSPS from Enamine or other collections can lead to a higher rate in CPA. However, we are aware that such a strategy may oppose the often-favored prioritization of lead-like compounds (with MW < 400) for screening campaigns to enable robust hit-to-lead optimization.

Given the sheer size of the Enamine library, there are still ample numbers of compounds that already meet the criteria mentioned above. That is, among the PNPs in the Enamine library alone, there are already 285 000 compounds with an nSPS ≥ 20, *i.e.* in the range that was identified as characteristic for compound classes enriched in bioactivity. It is to be expected that other vendors will also provide PNPs with these structural parameters. Our findings suggest that for future compound collection design principles of compound selection for hit-finding should include PNP-character and enrichment in bioactivity, and, thereby, extend beyond the rules of lead- or drug-likeness.^38,39^ Considering the currently available small molecule collections alone, if desired, a PNP screening library that promises to yield numerous hits with novel bioactivity is, in principle, readily available.

## Conclusions

Pseudo-natural products combine fragments derived from NPs in ways not accessible to current biosynthesis pathways and expand coverage of NP-like chemical space not populated by NPs, while pertaining their properties, in particular biological relevance. PNP collections are typically synthesized in multi-step reaction sequences which frequently include asymmetric complexity-generating transformations often forming multiple stereogenic centers. Investigation of pseudo-NP collections may lead to the discovery of novel chemotypes for unexpected targets or the identification of unprecedented bioactivities.

PNPs have been synthesized and investigated for bioactivity before, probably driven by intuitive inclusion of NP structures in compound library design, and they define a major fraction of currently known bioactive compounds. They are strongly enriched in compounds in phases 1–4, *i.e.* in clinical exploration and in the market.^16^ In light of this broad historical use and success, rapid access to PNPs, preferably without the need to develop multi-step complexity-generating asymmetric transformations, is of high relevance to future medicinal chemistry and chemical biology programs.

We have developed a strategy that gives rapid access to a large number of structurally different PNPs from commercial sources. To this end, we developed an improved version of the natural product fragment combination (NPFC) tool which identifies and classifies PNPs by identifying NP fragments and their combination types in chemical structures. Using this tool, we analyzed the full library of 3.5 M synthetic small molecules and screening libraries, which are available and widely sourced by the scientific community from Enamine for PNP content, as a proxy for potential future bioactive compounds. For possible correlation of structural complexity with degree of bioactivity, the spatial complexity of the Enamine library was assessed using the normalized spatial score (nSPS). Bioactivity of a selected subset of PNPs obtained from Enamine was determined in the unbiased morphological cell painting assay (CPA), and compared to the performance of a PNP collection synthesized in the course of research projects aimed at the establishment of the PNP principle. The analysis revealed that 32% of the Enamine library are PNPs with predominant fragment composition and combinations that resemble PNPs identified in the most recently released ChEMBL 32 dataset. Enamine-PNPs contain a significant number of compounds with unexpected and probably new bioactivity.

Cluster sub-profile analysis of the CPA data recorded for the active Enamine compounds revealed that at 30 μM, 106 out of 143 active compounds could not be assigned to any cluster (74%; 28 out of 44 active compounds at 10 μM (64%)). This finding suggests bioactivity that differs from the reference compounds employed for the cluster assignment and it was confirmed by means of UMAP analysis with the full morphological CPA profiles of these unassigned compounds.

These results highlight a qualitative advantage of PNPs, since they may be rich sources of compounds with unexpected and diverse, possible previously unidentified bioactivity.

The observed frequency of activity for the selected PNPs suggests that in the assembly of a PNP-library from commercial sources or through synthesis, a balanced approach should be used when considering the compound's physicochemical properties (in particular, nSPS and MW). Thus, if desired, a PNP screening library that promises to yield numerous hits with novel bioactivity is, in principle, readily available commercially without the need to design and execute laborious and time-consuming complexity-generating (asymmetric) syntheses programs.

## Materials and methods

### General information

The NPFC analyses were performed on an in-house cluster (4 nodes, 96 cores), using Python 3.9 and the NPFC tool in the latest version, available at https://github.com/jose-manuel/npfc.^22^ All further downstream processing and cheminformatics analyses were performed locally, using Python 3.11 and the RDKit, v2023.03.1.^40^ The normalized Spatial Score was calculated from the standardized SMILES using the code accompanying lit.^19^*Note: since v2023.09.1, SPS and nSPS are implemented directly in the RDKit.*

### Datasets

The ChEMBL structure dataset, v32, was downloaded from the FTP server (https://ftp.ebi.ac.uk/pub/databases/chembl/ChEMBLdb/releases/chembl_32/chembl_32.sdf.gz), the Enamine dataset containing 3.5 compounds encoded as SMILES was provided by Enamine.

### NPFC analysis

The NPFC tools requires three datasets for its identification of PNPs. (1) The set of natural product fragments. We used the Murcko scaffolds of the 2000 fragments published by Over *et al.* During the processing, the tool deduplicated the standardized fragment set by InChIKeys of the racemic scaffolds and deliberately excluded benzene, leaving 1673 fragments. (2) A library defining NPs. Here we used the Dictionary of Natural Products (DNP; 318 000 compounds). For (1) and (2), the same datasets were used as originally reported.^15^ (3) Finally, the dataset to be analyzed, here ChEMBL v32 and the Enamine library. Like (1), (2) and (3) were standardized and deduplicated by InChIKeys of the racemic structures. In addition, a deglycosylation step was applied, as originally reported.

The NPFC analyses for these two datasets were performed in the following way.

The structures of the original datasets were racemized, deglycosylated and standardized with the following command from the jupy_tools Python package: “stand_struct dataset.tsv fullrac -d --canon=legacy --deglyco”.^41^ The same original datasets were then also submitted to the analysis with the NPFC package.

Compared to the procedure described originally,^15^ all default configuration values were kept, except for the timeout parameter, which was increased to 50 s. In addition, the latest version of the NPFC tool was used, in which a bug was fixed which lead to an under-representation of PNP compounds. The fragment combination graphs for the PNPs and NPLs were extracted from the result files generated by the tool, merged by InChIKeys and analyzed for their fragment and connection type content. Finally, since the NPFC package applies some filters to the structures and deduplicates the dataset after standardization of the structures by InChIKeys before running the analysis (see above), the PNP status information and the fragment analysis was merged back to the standardized original full datasets, and all entries that were neither PNP, NPL or NP were assigned the status NonPNP.

### Selection of Enamine PNPs for CPA

Murcko scaffold SMILES and InChIKeys were generated for the 1.1 M PNPs in the Enamine dataset. The number of heavy atoms for each scaffold was calculated and the scaffold group sizes were determined for each Murcko scaffold by InChIKey. Groups with a scaffold of not exactly 17 heavy atoms or with less than 4 members were removed and 4 random members from 250 random scaffold groups were selected for measurement. Of these 1000 compounds, 875 were available in sufficient amount and purity (≥90% LCMS), and were provided by Enamine. The structures were proven to be PAINS-free, using an internal Pipeline Pilot protocol.

## Data availability

The following data is included in the ESI:† (1) the cell painting profiles containing 579 features for the 187 active measurements (44 active at 10 μM, 143 active at 30 μM), including structures of active compounds and structures encoded as SMILES. (2) The hundred most common fragment combinations of the Enamine PNP dataset.

## Author contributions

The manuscript was written through contributions of all authors. All authors have given approval to the final version of the manuscript.

## Conflicts of interest

O. O. G. and I. S. K. are employees/consulting scientists of Enamine Ltd.

## Supplementary Material

MD-015-D4MD00310A-s001

MD-015-D4MD00310A-s002

MD-015-D4MD00310A-s003

MD-015-D4MD00310A-s004

MD-015-D4MD00310A-s005
