# Supplementary material for: Identification of readily available pseudo-natural products
Source: RSC Med Chem. 2024 Jul 4;15(8):2709–17. doi: 10.1039/d4md00310a (PMC11324060; doi:10.1039/d4md00310a)
Supplement: MD-015-D4MD00310A-s004 [file MD-015-D4MD00310A-s004.pdf]

Electronic Supplementary Material (ESI) for RSC Medicinal Chemistry.
This journal is © The Royal Society of Chemistry 2024

Supporting_Information.docx:
Supporting information to the manuscript.
Contains figure S1 and the description of the Cell painting assay.

ds_enamine_pnp_cpa_actives.xlsx:
Cell painting data for 187 measurements that were found active.
44 measurements were active at 10µM, 143 were active at 30µM.

Columns:
Enamine_Id: Compound identifier (Enamine)
Compound_Id: Compound identifier (COMAS)
Well_Id: Unique identifier of a measurement in the dataset
Conc_uM: measurement concentration in µM
Induction: Cell painting induction value
Rel_Cell_Count: Cell count in percent, relative to DMSO controls
Toxic: Whether the compound is toxic (Rel_Cell_Count < 50) or not at the tested concentration
Cluster_High: The biological cluster with the highest similarity to this measurement,
using subprofile analysis
Cluster_Sim: The biosimilarity value to this cluster, in percent
Cluster_*: Individual biosimilarity to the given biological cluster,
using subprofile analysis, in percent
Smiles: SMILES encoding of the structure
SPS: Spatial score
nSPS: normalized spatial score
NumHA: number of heavy (non-hydrogen) atoms
PNP_Status
npfc_ctypes: fragment connection types found in the compound
npfc_num_frags: number of NP fragments found in the compound
Median_*: The 579 individual Cell painting feature values (Z-scores)

ds_enamine_pnp_cpa_actives_struct.xlsx:
Cluster assignments for 187 measurements that were found active.
44 measurements were active at 10µM, 143 were active at 30µM.

Columns:
Enamine_Id: Compound identifier (Enamine)
Conc_uM: measurement concentration in µM
Induction: Cell painting induction value
Rel_Cell_Count: Cell count in percent, relative to DMSO controls
Cluster: The assigned biological cluster, using subprofile analysis
A biosimilarity of >= 80% is required, otherwise the cluster is "not assigned"
Cluster_*: Individual biosimilarity to the given biological cluster,
using subprofile analysis, in percent

enamine_pnp_fcc.xlsx:
The hundred most common fragment combinations in the Enamine PNP set.

Columns:
fcc: Fragment combination classification. Has 3 parts: <Frag1_Id>[ctype]<Frag2_Id>
count: The number of occurrences in the data set
perc: Percentage of occurrence
frag1_smiles: Smiles of the first fragment
ctype: connection type (see manuscript)
frag2_smiles: Smiles of the second fragment
