# Supplementary material for: Identification of readily available pseudo-natural products
Source: RSC Med Chem. 2024 Jul 4;15(8):2709–17. doi: 10.1039/d4md00310a (PMC11324060; doi:10.1039/d4md00310a)
Supplement: MD-015-D4MD00310A-s005 [file MD-015-D4MD00310A-s005.pdf]

Electronic Supplementary Material (ESI) for RSC Medicinal Chemistry.
This journal is © The Royal Society of Chemistry 2024

Supporting Information

Identification of Readily Available Pseudo-Natural Products

Axel Pahl, Oleksandr O. Grygorenko, Ivan S. Kondratov, and Herbert Waldmann*

Table of contents

Supporting Figures....2

Cell Painting Assay....3

References....8

Supporting Figures

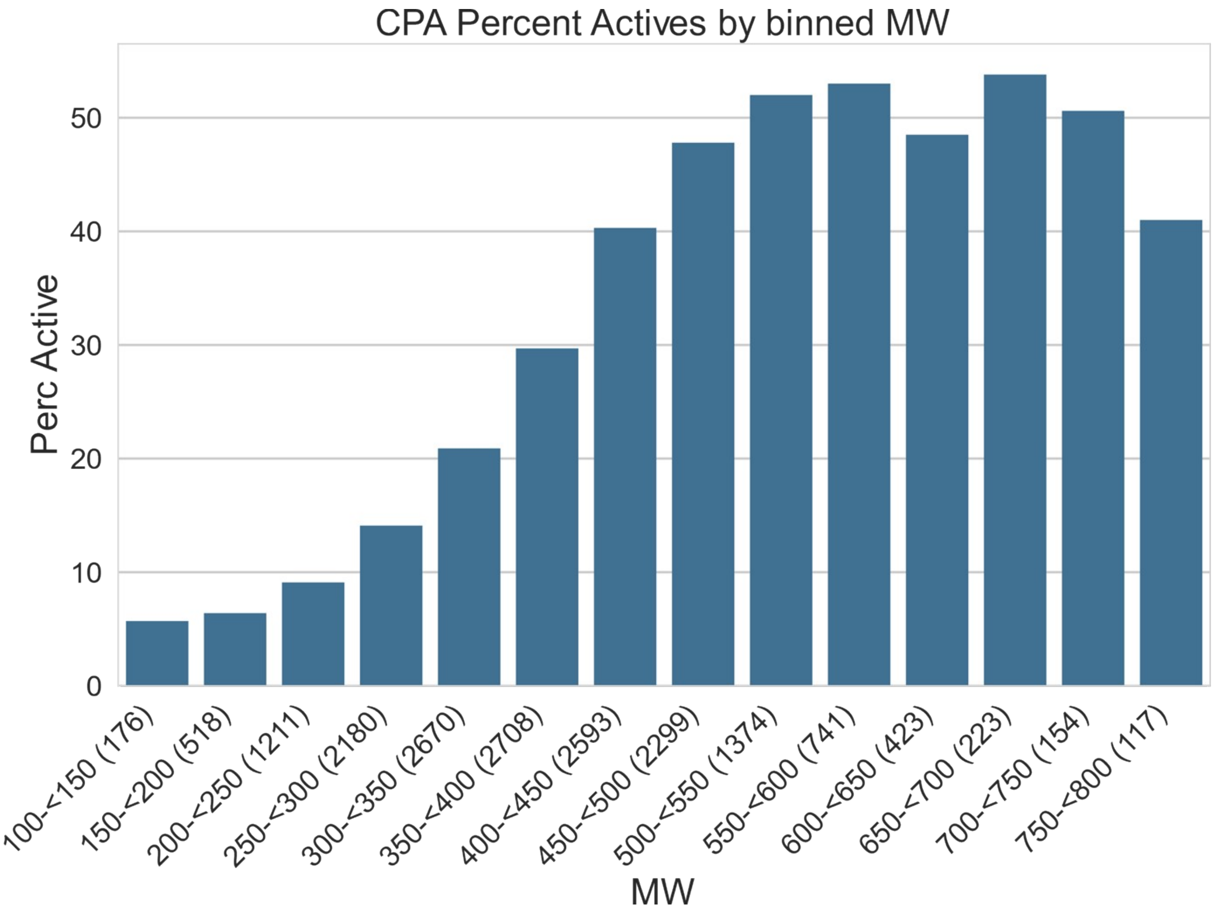

Figure S1. Ratio of active compounds in the cell painting assay (CPA) per binned molecular weight (MW). The plot shows the data for ~18,000 different compounds (references and internal campaigns), measured at standard concentration (10µM in general, 2µM for one 1536 plate of kinase inhibitors). The numbers in parentheses denote the number of compounds populating the given bin.

Cell Painting Assay

“The described assay follows closely the method described by Bray et al.[1]

Initially, 5 µl U2OS medium were added to each well of a 384-well plate (PerkinElmer CellCarrier-384 Ultra). Subsequently, U2OS cell were seeded with a density of 1600 cells per well in 20 µl medium. The plate was incubated for 10 min at the ambient temperature, followed by an additional 4 h incubation (37 °C, 5% CO2). Compound treatment was performed with the Echo 520 acoustic dispenser (Labcyte) at final concentrations of 10 µM, 3 µM or 1 µM. Incubation with compound was performed for 20 h (37 °C, 5% CO2). Subsequently, mitochondria were stained with Mito Tracker Deep Red (Thermo Fisher Scientific, Cat. No. M22426). The Mito Tracker Deep Red stock solution (1 mM) was diluted to a final concentration of 100 nM in prewarmed medium. The medium was removed from the plate leaving 10 µl residual volume and 25 µl of the Mito Tracker solution were added to each well. The plate was incubated for 30 min in darkness (37 °C, 5% CO2). To fix the cells 7 µl of 18.5 % formaldehyde in PBS were added, resulting in a final formaldehyde concentration of 3.7 %. Subsequently, the plate was incubated for another 20 min in darkness (RT) and washed three times with 70 µl of PBS. (Biotek Washer Elx405). Cells were permeabilized by addition of 25 µl 0.1% Triton X-100 to each well, followed by 15 min incubation (RT) in darkness. The cells were washed three times with PBS leaving a final volume of 10 µl. To each well 25 µl of a staining solution were added, which contains 1% BSA, 5 µl/ml Phalloidin (Alexa594 conjugate, Thermo Fisher Scientific, A12381), 25 µg/ml Concanavalin A (Alexa488 conjugate, Thermo Fisher Scientific, Cat. No. C11252), 5 µg/ml Hoechst 33342 (Sigma, Cat. No. B2261-25mg), 1.5 µg/ml WGA-Alexa594 conjugate (Thermo Fisher Scientific, Cat. No. W11262) and 1.5 µM SYTO 14 solution (Thermo Fisher Scientific, Cat. No. S7576). The plate is incubated

for 30 min (RT) in darkness and washed three times with 70 µl PBS. After the final washing step, the PBS was not aspirated. The plates were sealed and centrifuged for 1 min at 50 xg.

The plates were prepared in triplicates with shifted layouts to reduce plate effects and imaged using a Micro XL High-Content Screening System (Molecular Devices) in 5 channels (DAPI: Ex350-400/ Em410-480; FITC: Ex470-500/ Em510-540; Spectrum Gold: Ex520-545/ Em560-585; TxRed: Ex535-585/ Em600-650; Cy5: Ex605-650/ Em670-715) with 9 sites per well and 20x magnification (binning 2).

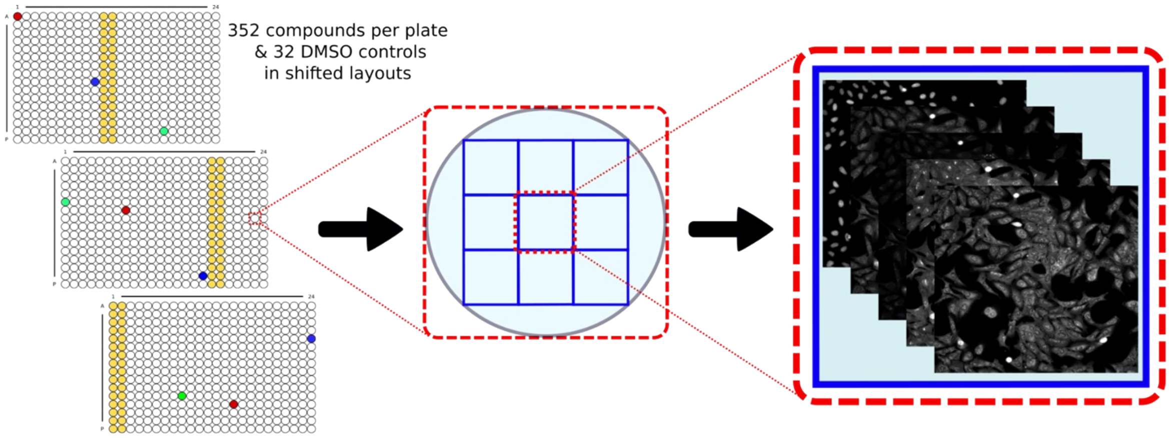

The generated images were processed with the *CellProfiler* package (https://cellprofiler.org/, version 3.0.0) on a computing cluster of the Max Planck Society to extract 1716 cell features per microscope site. The data was then further aggregated as medians per well (9 sites -> 1 well), then over the three replicates.

Further analysis was performed with custom *Python* (https://www.python.org/) scripts using the *Pandas* (https://pandas.pydata.org/) and *Dask* (https://dask.org/) data processing libraries as well as the *Scientific Python* (https://scipy.org/) package (separate publication to follow).

From the total set of 1716 features, a subset of highly reproducible and robust features was determined using the procedure described by Woehrmann et al.[2] in the following

way:

Two biological repeats of one plate containing reference compounds were analysed. For every feature, its full profile over each whole plate was calculated. If the profiles from the two repeats showed a similarity >= 0.8 (see below), the feature was added to the set.

This procedure was only performed once and resulted in a set of 579 robust features out of the total of 1716 that was used for all further analyses.

Determination of reproducible Features

1716 — *Determined by CellProfiler*

↓ — *Keep features that have a minimum correlation of 0.80 between repeats for all cpds.*

579 — *Final set of relevant features. Used for all further analyses*

The phenotypic profiles were compiled from the Z-scores of all individual cellular features, where the Z-score is a measure of how far away a data point is from a median value.

Specifically, Z-scores of test compounds were calculated relative to the Median of DMSO controls. Thus, the Z-score of a test compound defines how many MADs (Median Absolute Deviations) the measured value is away from the Median of the controls as illustrated by the following formula:

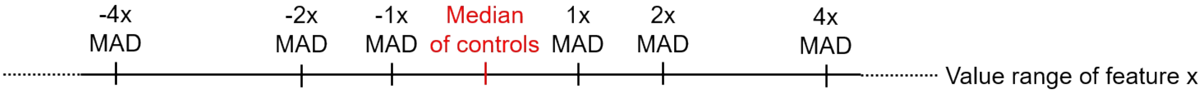

$$z - score = \frac{value_{meas.} - Median_{Controls}}{MAD_{Controls}}$$

The phenotypic compound profile is then determined as the list of Z-scores of all features for one compound.

In addition to the phenotypic profile, an induction value was determined for each compound as the fraction of significantly changed features, in percent:

$$Induction\ [\%] = \frac{number\ of\ features\ with\ abs.\ \ values\ >\ 3}{total\ number\ of\ features}$$

Similarities of phenotypic profiles (termed *Biosimilarity*) were calculated from the correlation distances (CD) between two profiles (https://docs.scipy.org/doc/scipy/reference/generated/scipy.spatial.distance.correlation.html):

$$CD = 1 - \frac{(u - \bar{u})\ \cdot\ (v - \bar{v})}{\|(u - \bar{u})\|_2 \|(v - \bar{v})\|_2}$$

where $\bar{x}$ is the mean of the elements of x, $x \cdot y$ is the dot product of x and y, and $\|x\|_2$is the Euclidean norm of x:

$$\|x\|_2 = \sqrt{x_1^2 + x_2^2 + \ldots + x_n^2}$$

The Biosimilarity is then defined as:

$$Biosimilarity = 1 - CD$$

Biosimilarity values smaller than 0 are set to 0 and the Biosimilarity is expressed in percent (0-100).

An example for two compounds with highly similar profiles (96% Biosimilarity):

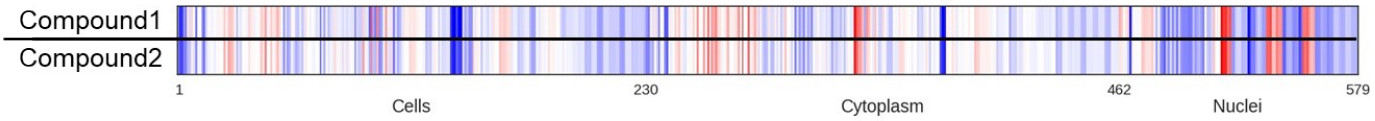

An example for two compounds with low similarity profiles (0% Biosimilarity):

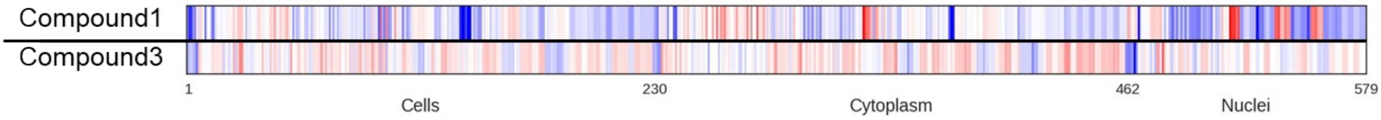

Each colored band represents one Z-score of a feature.

In addition to calculating biosimilarity between the full morphological profiles of two Cell painting measurements, Pahl et al. developed an approach to assign similarity to biological clusters by comparing sub-profiles.[3]

In essence, a set of 12 biological clusters was defined from Cell painting measurements with confirmed activity on these clusters. By considering only the features with similar values from the group of measurements for each cluster, a representative median profile was calculated for each cluster. These representative median profiles are of different length and shape for each cluster.

By comparing the median cluster profiles to the matching sub-profiles of measured compounds (only profiles of the same length can be compared for similarity), a biosimilarity to each cluster can be calculated.

The figure shows the list of currently identified clusters and their median profiles:

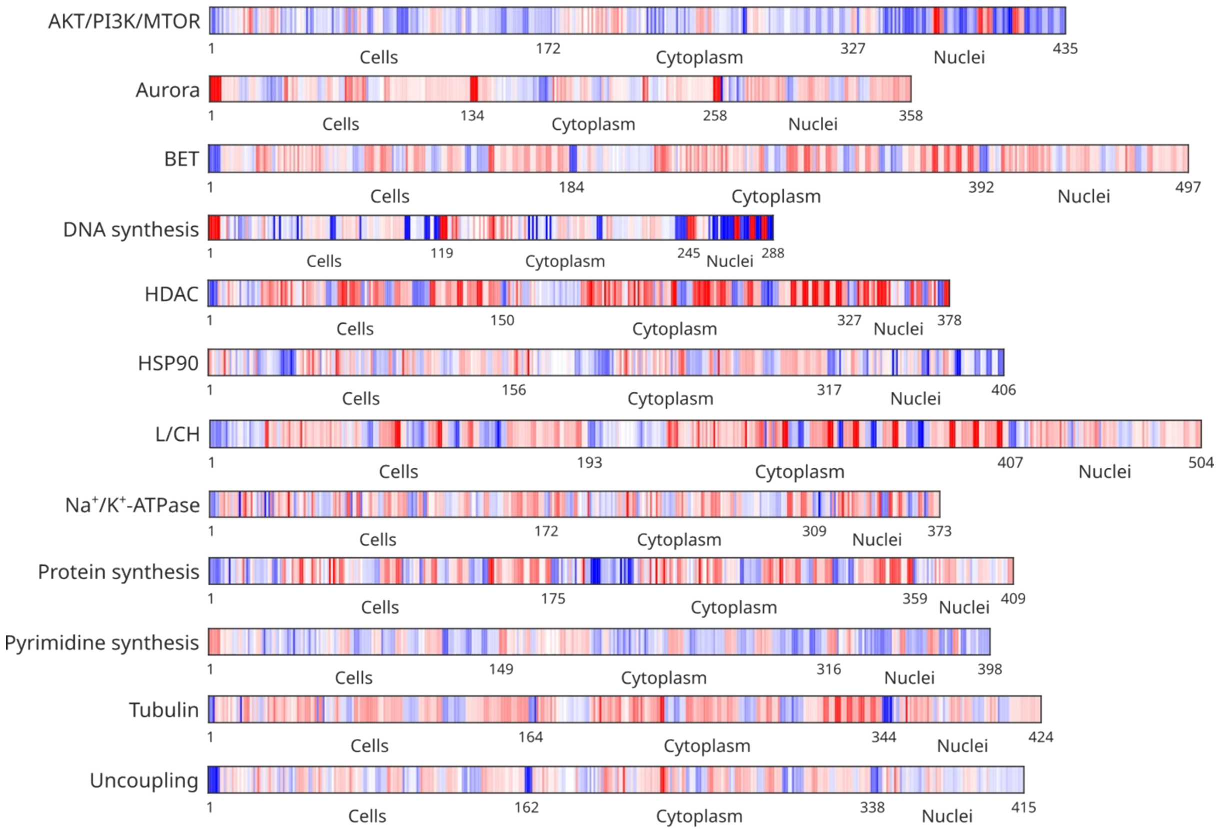

”

References

(1) Bray, M.-A.; Singh, S.; Han, H.; Davis, C. T.; Borgeson, B.; Hartland, C.; Kost-Alimova, M.; Gustafsdottir, S. M.; Gibson, C. C.; Carpenter, A. E. Cell Painting, a High-Content Image-Based Assay for Morphological Profiling Using Multiplexed Fluorescent Dyes. *Nat. Protoc.* **2016**, *11* (9), 1757–1774. https://doi.org/10.1038/nprot.2016.105.

(2) Woehrmann, M. H.; Bray, W. M.; Durbin, J. K.; Nisam, S. C.; Michael, A. K.; Glassey, E.; Stuart, J. M.; Lokey, R. S. Large-Scale Cytological Profiling for Functional Analysis of Bioactive Compounds. *Mol. Biosyst.* **2013**, *9* (11), 2604. https://doi.org/10.1039/c3mb70245f.

(3) Pahl, A.; Schölermann, B.; Lampe, P.; Rusch, M.; Dow, M.; Hedberg, C.; Nelson, A.; Sievers, S.; Waldmann, H.; Ziegler, S. Morphological Subprofile Analysis for Bioactivity Annotation of Small Molecules. *Cell Chem. Biol.* **2023**, *30* (7), 839-853.e7. https://doi.org/10.1016/j.chembiol.2023.06.003.
